# Artificial Intelligence in the Diagnosis and Imaging-Based Assessment of Pelvic Organ Prolapse: A Scoping Review

**DOI:** 10.3390/medicina61081497

**Published:** 2025-08-21

**Authors:** Marian Botoncea, Călin Molnar, Vlad Olimpiu Butiurca, Cosmin Lucian Nicolescu, Claudiu Molnar-Varlam

**Affiliations:** 1 Faculty of Medicine, George Emil Palade University of Medicine, Pharmacy, Sciences and Technology of Targu Mures, 540139 Targu Mures, Romania; marian.botoncea@umfst.ro (M.B.); vlad.butiurca@umfst.ro (V.O.B.);; 2 General Surgery Clinic No.1, County Emergency Clinical Hospital of Targu-Mures, 540136 Targu Mures, Romania; 3 Obstetrics and Gynecology Clinic No. 1, County Emergency Clinical Hospital of Targu-Mures, 540136 Targu Mures, Romania

**Keywords:** pelvic organ prolapse, artificial intelligence, deep learning, medical imaging, convolutional neural networks, vision transformers, ultrasound, magnetic resonance imaging, anatomical segmentation

## Abstract

*Background and Objectives*: Pelvic organ prolapse (POP) is a complex condition affecting the pelvic floor, often requiring imaging for accurate diagnosis and treatment planning. Artificial intelligence (AI), particularly deep learning (DL), is emerging as a powerful tool in medical imaging. This scoping review aims to synthesize current evidence on the use of AI in the imaging-based diagnosis and anatomical evaluation of POP. *Materials and Methods*: Following the PRISMA-ScR guidelines, a comprehensive search was conducted in PubMed, Scopus, and Web of Science for studies published between January 2020 and April 2025. Studies were included if they applied AI methodologies, such as convolutional neural networks (CNNs), vision transformers (ViTs), or hybrid models, to diagnostic imaging modalities such as ultrasound and magnetic resonance imaging (MRI) to women with POP. *Results*: Eight studies met the inclusion criteria. In these studies, AI technologies were applied to 2D/3D ultrasound and static or stress MRI for segmentation, anatomical landmark localization, and prolapse classification. CNNs were the most commonly used models, often combined with transfer learning. Some studies used hybrid models of ViTs, demonstrating high diagnostic accuracy. However, all studies relied on internal datasets, with limited model interpretability and no external validation. Moreover, clinical deployment and outcome assessments remain underexplored. *Conclusions*: AI shows promise in enhancing POP diagnosis through improved image analysis, but current applications are largely exploratory. Future work should prioritize external validation, standardization, explainable AI, and real-world implementation to bridge the gap between experimental models and clinical utility.

## 1. Introduction

Medical imaging plays an important role in gynecology, providing critical insights into uterine, ovarian, and pelvic floor conditions. Ultrasound is widely used due to its accessibility, affordability, and capacity for dynamic, real-time assessments [1]. Ultrasound is the primary imaging modality for evaluating uterine fibroids, adnexal masses, and intracavitary abnormalities, including those encountered in perimenopausal and postmenopausal women [2]. Magnetic resonance imaging (MRI) complements ultrasound by offering superior soft-tissue contrast and multiplanar visualization, which is particularly valuable in complex cases that require precise anatomical delineation or surgical planning [3,4].

Within this broader context, pelvic organ prolapse (POP) is a medical condition in which one or more of the following anatomical structures experiences a downward movement: the anterior vaginal wall, posterior vaginal wall, uterus (more specifically, the cervix), or apex of the vagina (the vaginal vault or cuff scar after hysterectomy) [5]. An accurate POP diagnosis is essential because it may influence treatment options, including surgical and non-surgical alternatives [6]. POP often involves more than one compartment. Therefore, imaging plays a vital role in assessing pelvic floor disorders [3].

Ultrasound, particularly transperineal and endovaginal approaches, is commonly used to assess pelvic floor integrity and dynamic function [7]. Meanwhile, static and stress MRI enables detailed visualizations of prolapse severity, organ relationships, and supportive structures. These modalities are increasingly employed not only to confirm clinical findings but also to guide surgical interventions through landmark localization, compartment classification, and anatomical segmentation [8].

Despite the utility of these imaging techniques, challenges remain. Diagnostic accuracy can be affected by patient-related variability such as bladder filling or physical effort during Valsalva [9], as well as by interobserver differences in interpreting results [10]. These factors create an urgent need for more standardized and reproducible imaging approaches.

Artificial intelligence (AI), particularly deep learning (DL), has emerged as a promising solution to these challenges. AI algorithms have demonstrated the capacity to automate complex tasks such as image segmentation, landmark detection, and multi-compartment prolapse classification, with the potential to reduce observer dependency and improve diagnostic consistency [11,12].

AI typically employs machine learning (ML) techniques to build models that mimic clinical decision-making [11,12]. Convolutional neural networks (CNNs), a deep learning (DL) mechanism used for image data analysis, are a common form of AI in medical imaging. CNNs can be used for classification and segmentation problems because they automatically learn features’ spatial hierarchies [13]. Encoder–decoder networks are particularly well-suited for medical image analysis tasks such as anatomical landmark recognition and organ segmentation. These networks consist of two parts: an encoder that compresses the input image into a lower-dimensional feature representation and a decoder that reconstructs the original spatial dimensions while making pixel-wise predictions [14]. Vision transformers (ViTs) have recently shown promise in interpreting multi-dimensional medical images using attention-based mechanisms [15]. Occasionally, deep learning models are integrated with gradient boosting frameworks such as XGBoost to enhance diagnostic performance via structured data analysis [16].

This scoping review aims to offer a comprehensive overview of current state-of-the-art AI-based methodologies for diagnosis and anatomic imaging evaluations of POP. This study highlights performance outcomes, clinical applicability, and research gaps that must be addressed to enable broader clinical adoption.

## 2. Materials and Methods

### 2.1. Study Protocol

This is a scoping review, a method used to assess evidence from related studies [17,18]. Although there is no consensus on the purpose or definition of a scoping review, most interpretations describe it as a methodology aimed at summarizing evidence to convey the importance of the subject [19]. Unlike systematic reviews [20], scoping reviews do not rigorously assess the quality of studies; instead, they typically employ broader research questions or inclusion/exclusion criteria, may not require data extraction, and often provide a more qualitative discussion of the results [17,18,19].

This review was conducted following the PRISMA Extension for Scoping Reviews (PRISMA-ScR) guidelines [21]. A comprehensive search of the medical literature was performed across three databases (PubMed, Scopus, and Web of Science), covering articles published from 1 January 2020 to 1 April 2025.

The protocol used for this scoping review was registered and can be accessed online at the following address: https://osf.io/fuzy9/?view_only=40bc8a5559f34e4e88e86e16e529b9b7 40bc8a5559f34e4e88e86e16e529b9b7 (accessed on 10 June 2025).

### 2.2. Eligibility Criteria

The eligibility criteria were developed using the Population–Concept–Context (PCC) framework recommended by the Joanna Briggs Institute for scoping reviews [22]. These criteria include the following: population (women with POP evaluated by imaging), concept (application of AI in diagnostic imaging of POP), and context (clinical and research settings employing imaging modalities such as ultrasound, CT, and MRI for POP assessment).

This scoping review considered original articles that employed AI methods such as ML, DL, or CNNs for diagnosing or evaluating POP images. Articles had to be written in English and include only human participants reporting outcomes related to image segmentation, POP classification, anatomical structure analysis, or predictive modeling. We excluded animal studies, reviews, editorials, conference abstracts, and letters to the editor.

### 2.3. Search Methodology

A combination of keywords and Medical Subject Headings (MeSH) such as “pelvic organ prolapse” [MeSH] OR “pelvic organ prolapse” [All Fields] OR POP AND “artificial intelligence” [MeSH] OR “machine learning” OR “deep learning” OR “neural network” OR “radiomics” AND “diagnosis” OR “assessment” OR “imaging” OR “MRI” OR “ultrasound” OR “CT”, along with related terms, was used. Articles were extracted from each database using a customized search plan, and additional sources were identified by manually screening the reference lists.

For PubMed, the full search strategy was as follows:

“((“Pelvic Organ Prolapse” [MeSH Terms] OR “Pelvic Organ Prolapse” [Title/Abstract] OR “Pelvic Floor Disorders” [MeSH Terms] OR “Pelvic Floor Disorders” [Title/Abstract] OR “pelvic floor dysfunction” [Title/Abstract])

AND

(“Artificial Intelligence” [MeSH Terms] OR “Artificial Intelligence” [Title/Abstract] OR “Machine Learning” [MeSH Terms] OR “Machine Learning” [Title/Abstract] OR “Deep Learning” [Title/Abstract] OR “neural networks, computer” [MeSH Terms] OR “neural networks” [Title/Abstract] OR “convolutional neural network” [Title/Abstract])

AND

(“Diagnosis” [MeSH Terms] OR “Diagnosis” [Title/Abstract] OR “Diagnostic Imaging” [MeSH Terms] OR “Diagnostic Imaging” [Title/Abstract] OR “Ultrasonography” [MeSH Terms] OR “ultrasound” [Title/Abstract] OR “Magnetic Resonance Imaging” [MeSH Terms] OR “Magnetic Resonance Imaging” [Title/Abstract] OR “MRI” [Title/Abstract])

AND

2010/01/01:2025/04/01 [Date-Publication])

AND ((y_5 [Filter]) AND (humans [Filter]) AND (English [Filter]))”.

For Scopus, the following query was used:

“(TITLE-ABS-KEY (“pelvic organ prolapse” OR “pelvic floor disorders” OR “pelvic floor dysfunction”))

AND

(TITLE-ABS-KEY (“artificial intelligence” OR “machine learning” OR “deep learning” OR “neural networks” OR “convolutional neural network”))

AND

(TITLE-ABS-KEY (“diagnosis” OR “diagnostic imaging” OR “ultrasound” OR “magnetic resonance imaging” OR “MRI”)) AND (LIMIT-TO (LANGUAGE, “English”))”.

For Web of Science search strategy was as follows:

“TS = (“pelvic organ prolapse” OR “pelvic floor disorders” OR “pelvic floor dysfunction”)

AND

TS = (“artificial intelligence” OR “machine learning” OR “deep learning” OR “neural networks” OR “convolutional neural network”)

AND

TS = (“diagnosis” OR “diagnostic imaging” OR “ultrasound” OR “magnetic resonance imaging” OR “MRI”)”.

### 2.4. Screening and Eligibility Assessment

Two reviewers evaluated each title and abstract independently. Full-text papers were obtained for studies that met the eligibility criteria or in cases where eligibility was uncertain. Disagreements were settled through discussion or by consulting a third member of the research team.

### 2.5. Data Charting and Extraction

Data were extracted using a standardized charting form created by the study team to facilitate data retrieval. The retrieved data included the publication year, country of origin, study design, sample size, AI technique, imaging modality, clinical application, and key performance measures. Both tabular and narrative summaries of the results featured descriptive language. The methodological and clinical variability of the included studies hindered any quantitative synthesis or meta-analysis. M.B. and V.O.B. reviewed the data extraction. MC was consulted if any disagreements remained unresolved.

In addition to general methodological characteristics, we also charted technical elements of the AI training pipelines reported in the included studies. These details included the nature of the input datasets (e.g., MRI or ultrasound images), whether annotations were manually performed by clinical experts, the use of specific annotation tools, and any data augmentation strategies described. Capturing these aspects was important for understanding the transparency, reproducibility, and robustness of the models. The extracted information is summarized in Table 1, providing a comparative overview of the AI training workflows across the studies.

### 2.6. Level of Evidence Assessment

In addition to data collection, we conducted an evidence-level assessment to provide insight into the methodological robustness of the included studies. The Oxford Centre for Evidence-Based Medicine (OCEBM) 2011 Levels of Evidence tool was used to classify the articles used in this scoping review [29]. This framework categorizes studies based on their design, with Level 1 representing the highest quality, such as randomized trials, and Level 5 representing expert opinions or mechanism-based reasoning. The assigned levels for each study are summarized in Table 2.

## 3. Results

### 3.1. Study Selection

**Figure 1:**
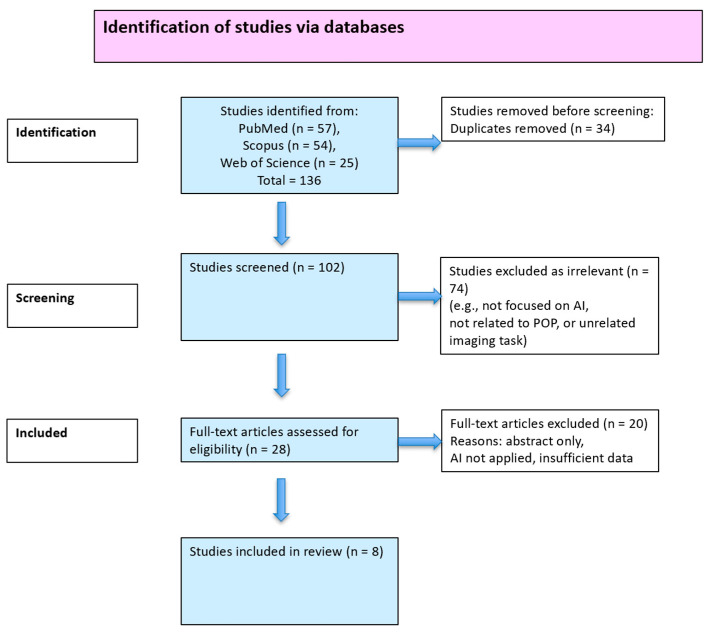
PRISMA-ScR flow diagram for the identification and selection of studies.

The preliminary search of the database resulted in 136 records sourced from PubMed (57), Scopus (54), and Web of Science (25). After removing duplicates, 102 records were retained and screened based on their titles and abstracts. Then, 28 full-text articles were assessed for eligibility, with 8 meeting the inclusion criteria and being included in the final synthesis. Of the 20 excluded full-text articles, the most common reasons were as follows: lack of AI application to imaging data, insufficient methodological or diagnostic detail, irrelevance to POP, and publication type (e.g., review, editorial, or abstract only). The study selection process is illustrated in the PRISMA-ScR flow diagram (Figure 1).

### 3.2. Study Characteristics

The eight studies included in this analysis were published between 2021 and 2025. They featured a diverse array of designs, including prospective observational studies, retrospective cohort studies, technical feasibility studies, and research focused on model development or validation [8,22,23,24,25,26,27,28]. Sample sizes ranged from 64 to 1805 participants, all of whom were female patients with clinically diagnosed POP. Most studies took place at single centers, primarily in China [8,22,23,24,25,26,27,28]. The studies employed AI techniques for various imaging tasks, including segmenting pelvic floor structures, identifying anatomical landmarks, classifying the prolapse types, and predicting prolapse severity. The imaging modalities included 2D ultrasound, 3D ultrasound, and MRI, including stress MRI [8,22,23,24,25,26,27,28]. Table 3 and Table 4 summarize all the relevant information regarding imaging modalities, AI methods, performance metrics, and study types.

### 3.3. Imaging Modalities and AI Approaches

#### 3.3.1. Ultrasound

The four ultrasound-based studies employed both 2D and 3D imaging techniques to assess POP using DL models [22,24,25,28]. Among these, CNNs were the most frequently used, often integrated with transfer methods such as ResNet-18 or VGG-16 [24]. Studies using 2D transperineal ultrasound videos demonstrated the highest diagnostic performance, likely due to consistent acquisition parameters and well-annotated datasets. For example, models built on 2D ultrasound with hybrid CNN-XGBoost approaches achieved superior precision and recall along with low inference times, indicating feasibility for integration into clinical workflows [16]. In contrast, 3D ultrasound [22,25] studies, while offering volumetric anatomical data, faced more variability in segmentation quality and often lacked comprehensive reporting of diagnostic metrics. This discrepancy may be due to challenges associated with labeling volumetric data and greater inter-patient anatomical variability in 3D datasets [22,25].

#### 3.3.2. MRI

MRI-based approaches in the included studies utilized both static and stress MRI protocols to explore segmentation, landmark localization, and multi-label classification tasks. These studies generally demonstrated high diagnostic potential, particularly when employing attention-based architectures such as vision transformers (ViTs) [15]. Such models captured spatial dependencies effectively and showed good concordance with clinical assessments, as reflected by substantial kappa values and AUCs [8,23,26,27]. However, differences in performance across MRI studies were evident and may be attributed to variations in sample size, labeling strategies, and whether stress imaging was employed. Additionally, several studies focused exclusively on segmentation without linking anatomical delineation to diagnostic accuracy, limiting interpretability for clinical application [23,26]. The lack of standardization in MRI acquisition parameters and the absence of external validation further contributed to the variability in reported outcomes.

#### 3.3.3. AI Methodologies

In the studies examined, the methodologies related to AI displayed varying degrees of complexity and breadth, with emphasis on DL-based strategies. Convolutional neural networks (CNNs) were the most used architecture, appearing in five studies [8,24,25,26,28], frequently enhanced through transfer learning using pretrained models such as AlexNet, VGG-16, or ResNet-18 [24,25]. Hybrid models, such as a CNN combined with XGBoost, represented efforts to integrate robust feature extraction with structured classification [16]. Encoder–decoder architecture was widely adopted in segmentation and landmark localization tasks, particularly in stress MRI datasets [27]. More recently, vision transformers (ViTs) have shown promise in analyzing multi-sequence imaging due to their attention-based mechanisms, indicating a shift toward more context-aware AI frameworks [15]. Despite these methodological advances, several limitations were consistent across studies. All models were trained and validated on internal datasets, with no external validation reported. Descriptions of preprocessing steps and augmentation strategies were sparse or absent. Furthermore, explainability tools such as Grad-CAM or SHAP, which could enhance model transparency and clinical trust, were rarely employed or discussed [30,31].

## 4. Discussion

### 4.1. Summary of the Main Findings

This scoping review summarizes data on the use of AI in the imaging-based anatomical evaluation and diagnosis of POP. The papers included in this review suggest that AI models, particularly those using DL, perform well in identifying anatomical landmarks, segmenting pelvic structures, and classifying prolapse compartments. Nonetheless, even with these technological advancements, methodological and translational obstacles must be resolved to enable clinical application. Most studies used CNNs for image classification and segmentation, a reasonable choice considering that CNNs were able to recognize spatial patterns [8,24,25,26,28].

### 4.2. Advances in AI Architectures

Some studies have improved these models using transfer learning [8,24], thus achieving pretrained architecture. ResNet is one example, as this neural network allows rapid adaptation to medical databases [8,24]. Newer models, including ViTs, have emerged, proving their ability to analyze multi-sequence MRI and suggesting a future transition toward more context-aware AI frameworks in pelvic imaging [23]. Hybrid technologies, such as CNN XGBoost combinations, have also proven useful, indicating continued efforts to integrate feature extraction and effective classification [28].

While most studies in this review focused on deep learning, traditional machine learning (ML) techniques also play a relevant role in POP assessment. Algorithms such as support vector machines (SVMs), random forests, and gradient boosting (e.g., XGBoost) have been used to classify prolapse compartments or support segmentation workflows when combined with engineered features [16]. ML methods often require smaller datasets than DL models and can be more interpretable, making them advantageous in settings where data quantity or labeling is limited [13]. Additionally, ML can integrate structured clinical or demographic data alongside imaging features, potentially enhancing predictive models for symptom severity or surgical outcomes [11,12]. As AI tools evolve, combining ML and DL in hybrid models may offer improved performance and clinical applicability, especially when transparency and explainability are priorities [16].

### 4.3. Clinical Relevance and Integration

These systems have a wide range of clinical applications, including compartment-specific POP classification, the automated segmentation of pelvic structures, and landmark localization [8,22,23,24,25,26,27,28]. These functions are essential for diagnosis and surgical planning. However, while imaging and AI represent objective tools for anatomical assessment, clinical evaluation remains central to the diagnosis and management of POP. Physical examination techniques, such as the pelvic organ prolapse quantification system (POP-Q) [32], provide functional and positional context that imaging alone may not capture. Moreover, symptoms often correlate poorly with anatomical findings, reinforcing the importance of a comprehensive clinical evaluation. AI-assisted imaging should, therefore, be understood as a complementary tool that enhances, but does not substitute clinical judgment. Integrating AI outputs with patient history, physical findings, and quality-of-life measures will be essential to ensure meaningful and patient-centered care.

Furthermore, imaging data may contribute to more tailored therapeutic strategies. For example, patients with AI-confirmed multi-compartment prolapse or associated anatomical descent may benefit from robotic or mini-laparoscopic colposacropexy [33], while concurrent lower urinary tract symptoms might prompt consideration of neuromodulatory treatments such as sacral nerve stimulation [34]. Given that urinary incontinence and prolapse frequently coexist and can substantially affect patients’ physical and emotional well-being [35,36], the diagnostic insights provided by AI could help support more comprehensive and individualized management. The psychological dimension of POP, including embarrassment, altered self-image, and sexual dysfunction, further underscores the value of early and accurate diagnosis within a multidisciplinary care framework [37].

### 4.4. Unresolved Technical and Methodological Issues

However, few studies have investigated multi-label or multi-compartment models, which would capture the intricacies of real-world POP scenarios more effectively [20]. Moreover, segmentation and localization tasks have generally focused on anatomical precision rather than direct diagnosis, indicating a disconnect between technical capabilities and clinical outcomes [23,27].

Despite the high quality of the models, the lack of external validation remains a problem. All surveyed studies relied on internal datasets, with no reviews of independent cohorts or across institutions [23,25,28]. This situation reduces the generalizability and reproducibility of the findings and raises concerns about overfitting, particularly in studies with smaller sample sizes. Furthermore, crucial features such as model interpretability, preprocessing approaches, and data augmentation have been reported in an inconsistent manner [23,24].

Furthermore, the lack of explainable AI solutions, such as SHapley Additive exPlanations (SHAP) or gradient-weighted class activation mapping, undermines clinician confidence and prevents potential AI’s inclusion into conventional processes. Notably, explainable AI solutions aim to explain the decision-making process of an ML or CNN [30,31].

### 4.5. Challenges in Imaging and AI Performance

Direct comparisons between models are further complicated by the diversity of imaging procedures, which range from 2D/3D ultrasound to static and stress MRI [8,22,23,24,25,26,27,28]. Studies using ultrasound have benefited from real-time data collection [22,24,25,28], whereas MRI offers greater anatomical information [8,23,26,27].

Imaging a POP presents distinct challenges due to the dynamic and multi-compartmental nature of pelvic anatomy [38]. This condition often involves varying degrees of descent across the anterior, apical, and posterior compartments, which may not be fully captured in static imaging or standard clinical exams [38]. Furthermore, differences in patient positioning (supine vs. upright), variability in strain effort during imaging, and the subtlety of certain anatomical shifts contribute to diagnostic uncertainty [39]. AI has the potential to address these limitations by providing consistent and objective landmark localization [27], automating multi-compartmental segmentation [23], and reducing interobserver variability. Deep learning algorithms, particularly when trained on diverse imaging modalities such as stress MRI or 3D ultrasound, could enhance anatomical detail recognition and facilitate a more accurate and reproducible assessment of pelvic floor disorders [22,25].

The choice of imaging modality influences the AI methodology, with transformer models and multi-label classifiers more commonly associated with MRI and CNNs chosen for ultrasound evaluations [8,24,27,28]. However, there remains no research on prospective deployment, clinical applicability, or patient outcomes following AI-assisted evaluations. Only Feng et al. investigated the viability of real-time applications in dynamic imaging, highlighting the critical need for implementation studies and prospective trials [26].

### 4.6. Limitations of the Study

While formal critical appraisal is not mandated in scoping reviews, several methodological limitations across the included studies warrant attention. Dataset sizes were generally small, with training often performed on fewer than 300 cases, increasing the risk of overfitting, particularly in deep learning applications [8,23,28]. Moreover, external validation was absent in all studies, raising concerns about model generalizability beyond the original clinical settings [22,23,24,25,26,27,28]. Many models reported high internal performance metrics (e.g., F1-scores > 90%). However, without independent testing, the robustness of these results remains uncertain. This limitation, coupled with the limited reporting of data augmentation strategies [23,26], underscores the need for cautious interpretation and highlights a broader challenge in AI-based diagnostic research on pelvic organ prolapse.

This scoping review also faces limitations due to the small number of eligible studies and their varying design, data, and outcome reporting. Some studies suffered from class imbalance due to the underrepresentation of posterior compartment prolapse, which may have biased performance metrics. Furthermore, few studies compared AI results with those of human expert evaluators, limiting clinical interpretability. The multiplicity of imaging modalities, AI techniques, and clinical objectives has also impeded a meta-analysis and a descriptive analysis.

### 4.7. Ethical, Regulatory, and Global Considerations

The protection of patient data is a key concern, given the highly personal nature of medical information. Thus, implementing strict data security measures is critical for retaining patient trust and adhering to legal standards [12]. The development and use of AI tools should align with data protection regulations, such as Europe’s General Data Protection Regulation (GDPR) [40]. Furthermore, future applications should consider algorithmic fairness and the medico-legal implications of diagnostic errors, ensuring that models are equitable, interpretable, and supported by clear accountability frameworks.

### 4.8. Implementation Challenges and Opportunities

The practical implementation of AI in pelvic organ prolapse assessment presents both promising opportunities and significant challenges. One potential avenue for application is the integration of AI tools into real-time imaging workflows, particularly in ultrasonography, where models with rapid inference speeds—such as those described by García-Mejido et al. [28]—could provide instant diagnostic feedback during clinical examinations. For MRI-based evaluations, AI can assist radiologists by automatically localizing pelvic landmarks and segmenting relevant structures, thereby reducing manual workload and improving consistency. Such systems could be embedded into Picture Archiving and Communication Systems (PACS) or integrated into radiology reporting software to support structured diagnostic interpretations.

However, the clinical adoption of these technologies also faces regulatory and operational barriers. To our knowledge, no AI models for POP assessment have obtained regulatory approval (e.g., Food and Drug Administration—FDA or European Medicines Agency—EMA), largely due to the absence of prospective validation studies and the insufficient reporting of algorithmic transparency and fairness. Furthermore, implementing AI in practice will require collaboration across disciplines, including radiologists, urogynecologists, and informatics teams, to ensure seamless workflow integration and interpretability.

In addition, data privacy regulations, such as the GDPR in Europe, impose strict constraints on model development and deployment, necessitating anonymization protocols and secure data handling pipelines [40].

The potential role of AI in low- and middle-income countries (LMICs) should also be considered. In settings where access to specialized imaging modalities such as MRI is limited, AI-enhanced ultrasonography could serve as a cost-effective, portable diagnostic solution for POP. Automated interpretation tools may help overcome the shortage of trained urogynecologists and radiologists, enabling earlier and more equitable diagnosis. However, the implementation of AI in these regions must account for barriers such as limited digital infrastructure, lack of local data for model training, and the need for regulatory and clinical validation in resource-constrained environments. Addressing these issues, particularly through explainable AI and adherence to reporting standards such as CLAIM and CONSORT-A, will be crucial for the safe, ethical, and effective clinical integration of AI in pelvic imaging [41,42].

### 4.9. Potential Bias and Reporting Gaps

In addition, potential publication bias must be considered. Studies reporting strong model performance or positive findings may be more likely to reach publication, while those with negative or inconclusive results may remain unpublished. This bias could lead to an overestimation of AI’s effectiveness in pelvic organ prolapse diagnosis and imaging. Furthermore, the exclusion of the non-English and gray literature may have limited the scope of included evidence.

### 4.10. Future Perspectives

Although performance data were extracted when available, the absence of standardization in reporting, especially for segmentation and localization tasks, hindered comparisons. Therefore, to more effectively incorporate AI into the diagnosis and assessment of POP, future research should focus on external and multicenter validation of the AI models; the creation of standardized, open-access imaging datasets; and the adoption of transparent reporting frameworks. In particular, adherence to tools such as the CLAIM checklist [41] and CONSORT-AI extension [42] is essential. These instruments can guide authors in reporting key technical, clinical, and validation components of AI research, thereby promoting replicability, clarity, and clinical relevance [41,42]. By implementing such structured frameworks, future studies can ensure a more robust methodology, enhance model interpretability, and build confidence among clinicians and regulators alike. The next step should be to conduct prospective studies to assess the impact of diagnostic accuracy on surgical planning and patient outcomes. To support the development of generalizable AI models, there is a critical need to generate larger, standardized, and multicenter imaging datasets specific to POP. These datasets should include diverse populations, multiple imaging modalities, and well-annotated ground truth labels to improve model training, validation, and benchmarking. Collaboration among multiple disciplines will help transform these technologies from experimental models to effective therapeutic tools.

## 5. Conclusions

This scoping review illustrates the growing relevance of AI in the imaging-based diagnosis and anatomical evaluation of pelvic organ prolapse (POP). Deep learning models, especially convolutional neural networks and transformer-based architectures, can attain high diagnostic accuracy, reliable segmentation, and landmark localization across various imaging modalities.

Incorporating AI into the regular evaluation of POP is likely to increase diagnosis accuracy, lower interobserver variability, and improve tailored treatment plans. Thus, future studies should focus on methodological rigor, standardization, and validation in real-world applications to ensure promising prototypes are transformed into useful tools.

## Figures and Tables

**Table 1:** Technical characteristics of AI training workflows in included studies.

Study (Author, Year)	Dataset Type	Annotation Method	Annotation Tool (If Stated)	Augmentation Reported	Notes on Interobserver Reliability
Wang et al., 2022 [8]	Labeled stress MRI (multi-label POP classification)	Manual expert labeling	Not reported	Yes, but no details	Not reported
Szentimrey et al., 2023 [22]	Labeled 3D ultrasound (mid-sagittal plane segmentation)	Manual segmentation	Not reported	Not stated	Not reported
Zhu et al., 2025 [23]	Labeled multi-sequence MRI for POP diagnosis	Manual annotation (details limited)	Not reported	Yes, geometric transforms	Not reported
Yang et al., 2025 [24]	Labeled ultrasound for anterior compartment POP	Manual expert annotations	Not reported	Yes, flipping, rotation	Not reported
Duan et al., 2021 [25]	Labeled ultrasound for POP identification	Manual annotation (POP stage)	Not reported	Yes, flipping, brightness	Not reported
Feng et al., 2020 [26]	Labeled MRI for pelvic floor segmentation	Manual delineation	Not reported	No	Not reported
Feng et al., 2021 [27]	Labeled stress MRI for landmark localization	Manual landmark placement	Not reported	Not stated	Not reported
García -Mejido et al., 2025 [28]	Ultrasound dataset labeled for POP compartments	Manual annotation by urogynecologists	Not reported	Yes, general augmentation	Not reported

**Table 2:** OCEBM levels of evidence for the included studies.

Study (First Author, Year)	Study Design	AI Focus	OCEBM Level of Evidence
García-Mejido et al., 2025 [28]	Prospective observational	2D ultrasound, CNN + XGBoost	Level 2
Yang et al., 2025 [24]	Retrospective cohort	2D ultrasound, DL architectures	Level 3
Duan et al., 2021 [25]	Retrospective comparative	3D ultrasound, DL classification	Level 3
Szentimrey et al., 2023 [22]	Technical segmentation	3D ultrasound, anatomical mapping	Level 4
Zhu et al., 2025 [23]	Model development + validation	MRI, vision transformer	Level 2
Feng et al., 2021 [27]	Feasibility study	Stress MRI, landmark localization	Level 4
Feng et al., 2020 [26]	Technical segmentation study	MRI, CNN	Level 4
Wang et al., 2022 [8]	Retrospective model development	Stress MRI, ResNet-50	Level 3

**Table 3:** Study characteristics.

Article	Modality	AI Method	Other Metrics	Article Type
Ultrasound Diagnosis of POP Using AI [28]	2D Ultrasound	CNN + XGBoost		Prospective Observational Study
Building a POP Diagnostic Model Using Vision Transformer [23]	MRI (multi-sequence)	Vision transformer	Kappa: 0.77	Model Development and Validation
Exploring the Diagnostic Value of PF Ultrasound via DL [25]	3D Ultrasound	CNN	Specificity: 84%	Comparative Study
Automated Segmentation of the Female Pelvic Floor (3D US) [22]	3D Ultrasound	Segmentation (DL)		Technical Segmentation Study
Combining Pelvic Floor US with DL to Diagnose Anterior Compartment POP [24]	2D Ultrasound	AlexNet/VGG-16/ResNet-18	Inference time: 13.4 ms	Retrospective Study
Conventional NN-Based Pelvic Floor Segmentation using MRI in POP [26]	MRI	CNN	No diagnostic metrics reported	Segmentation Feasibility Study
Feasibility of DL-Based Landmark Localization on Stress MRI [27]	Stress MRI	Encoder–decoder CNN	Localization error: 0.9 to 3.6 mm, time: 0.015 s	Feasibility Study
Multi-label Classification of POP Using Stress MRI with DL [8]	Stress MRI	Modified ResNet-50		Model Development and Validation

VGG = Visual Geometry Group; AlexNet = Deep convolutional neural network architecture for image classification; ResNet-18 = Residual Neural Network with 18 layers; XGBoost = eXtreme Gradient Boosting.

**Table 4:** Performance metrics.

Article	Accuracy	Recall	Precision	F1-Score	AUC
Ultrasound Diagnosis of POP Using AI [28]	98.31%	100%		98.18%	
Building a POP Diagnostic Model Using Vision Transformer [23]		0.76	0.86		0.86
Exploring the Diagnostic Value of PF Ultrasound via DL [25]	86%	89%			0.79
Automated Segmentation of the Female Pelvic Floor (3D US) [22]					
Combining Pelvic Floor US with DL to Diagnose Anterior Compartment POP [24]	93.53%				0.852
Conventional NN-Based Pelvic Floor Segmentation using MRI in POP [26]					
Feasibility of DL-Based Landmark Localization on Stress MRI [27]					
Multi-label Classification of POP Using Stress MRI with DL [8]		0.72	0.84	0.77	0.91

AUC = Area Under the Curve; Accuracy—proportion of correct predictions; Recall—proportion of true positives identified; Precision—proportion of relevant instances among the retrieved; F1-score = harmonic mean of precision and recall (means the model correctly identifies most prolapse cases without over-predicting false positives).

## Data Availability

No new data were created or analyzed in this study. Data sharing is not applicable to this article.

## Abbreviations

The following abbreviations are used in this manuscript:

POP	Pelvic organ prolapse
AI	Artificial intelligence
AUC	Area Under the Curve
CLAIM	Checklist for Artificial Intelligence in Medical Imaging
CNN	Convolutional neural network
CONSORT	Consolidated Standards of Reporting Trials
CT	Computer tomography
DE	Deep encoder (Encoder–Decoder)
DL	Deep learning
GDPR	General Data Protection Regulation
JBI	Joanna Briggs Institute
MRI	Magnetic resonance imaging
PCC	Population–Concept–Context
PRISMA-ScR	Preferred Reporting Items for Systematic Reviews and Meta-Analyses extension for Scoping Reviews
SHAP	Shapley Additive exPlanations
SVM	Support vector machine
VGG	Visual Geometry Group
ViT	Video transformer
ResNet-18	Residual Neural Network with 18 layers
XGBoost	eXtreme Gradient Boosting
PACS	Picture Archiving and Communication Systems
FDA	Food and Drug Administration
EMA	European Medicines Agency
POP-Q	Pelvic organ prolapse quantification system
